# Maternal preconception lipid profile and gestational lipid changes in relation to birthweight outcomes

**DOI:** 10.1038/s41598-019-57373-z

**Published:** 2020-01-28

**Authors:** Alaina M. Bever, Sunni L. Mumford, Enrique F. Schisterman, Lindsey Sjaarda, Neil J. Perkins, Nicole Gerlanc, Elizabeth A. DeVilbiss, Robert M. Silver, Keewan Kim, Carrie J. Nobles, Melissa M. Amyx, Lindsay D. Levine, Katherine L. Grantz

**Affiliations:** 1 Epidemiology Branch, Division of Intramural Population Health Research, Eunice Kennedy Shriver National Institute of Child Health and Human Development, 6710B Rockledge Drive, MSC 7004, Bethesda, MD 20892 United States; 2 The Prospective Group, 1655 Fort Myer Dr #700, Arlington, VA 22209 United States; 3 Department of Obstetrics and Gynecology, University of Utah, 30 North 1900 East, Salt Lake City, UT 84132 United States

**Keywords:** Biomarkers, Epidemiology

## Abstract

In 575 women with 1–2 prior pregnancy losses; total cholesterol, low-density lipoprotein cholesterol (LDL-C), high-density lipoprotein cholesterol (HDL-C), and triglycerides (TG) were evaluated preconception and throughout pregnancy to evaluate whether previously observed associations between third trimester maternal lipid profile and birthweight outcomes are driven by preconception lipids or lipid changes during pregnancy. Lipid trajectories were compared by pre-pregnancy body mass index (BMI) <25 or ≥25 kg/m^2^; logistic regression models evaluated preconception lipid concentration and change from preconception to 28 weeks with adjusted odds of large- or small-for-gestational age (LGA or SGA) neonate by BMI group. Preconception lipid concentrations and gestational lipid trajectories varied by BMI group (P < 0.001). Preconception lipids were not associated with LGA or SGA in either group. A 10 mg/dL increase in HDL-C change from preconception to 28 weeks was associated with decreased odds of LGA (odds ratio (OR) = 0.63, 95% confidence interval (CI): 0.46, 0.86) and 10 mg/dL increase in TG change associated with increased odds of LGA (OR = 1.05, 95% CI: 1.01, 1.1) overall. For ≥25 BMI only, 10 mg/dL increase in HDL-C change was associated with decreased SGA odds (OR = 0.35, 95% CI: 0.19, 0.64). Gestational lipid trajectories differed by BMI group and were differentially associated with birthweight outcomes, with HDL-C more strongly associated with healthy birthweight in women with BMI ≥25.

## Introduction

Pregnancies resulting in large-for-gestational age (LGA) or small-for-gestational age (SGA) neonates are associated with numerous adverse maternal and fetal outcomes^1–6^, including poor offspring cardiometabolic health in adulthood^7,8^. Extremely elevated lipid concentrations late in pregnancy have been associated with increased birthweight and macrosomia independent of body mass index (BMI) and glucose tolerance^9–14^, indicating that abnormal maternal lipids may be a risk factor for fetal overgrowth. Little is known about the relationship between preconception and gestational lipid profiles, or between preconception lipid profile and birthweight outcomes.

In non-pregnant populations, increased BMI is associated with unfavorable lipid profile characterized by low concentration of high-density lipoprotein cholesterol (HDL-C) and high concentrations of low-density lipoprotein cholesterol (LDL-C) and triglycerides (TG)^15^. During gestation, maternal lipid concentrations increase dramatically, with different gestational lipid trajectories based on maternal BMI^16,17^. However, no studies to our knowledge have investigated the relationship between differential lipid changes from preconception to late-pregnancy and birthweight outcomes, and the few studies on preconception lipid profile in relation to birthweight outcomes were limited to relatively healthy, low BMI cohorts^18,19^. It is unknown whether the association between late-pregnancy lipids and birthweight outcomes is explained by preconception lipid profile or a deviation from normal lipid changes during pregnancy.

The aims of this study were to analyze trajectories of lipid concentration from preconception throughout pregnancy by BMI group, and to investigate how preconception lipids and lipid changes from preconception to the time of lipid peak during gestation were related to birthweight outcomes. Effect modification by BMI was investigated because both preconception lipid profile and gestational lipid changes differ by BMI and may help explain the increased incidence of extreme birthweight outcomes in pregnancies complicated by obesity.

## Materials and Methods

### Study setting

The present study was a secondary analysis of the Effects of Aspirin in Gestation and Reproduction (EAGeR) trial, a block-randomized, double-blind, placebo-controlled trial evaluating the effect of preconception-initiated daily low-dose aspirin on live birth. Study design and inclusion and exclusion criteria have been previously reported in detail^20^. The trial enrolled 1,228 women with 1–2 prior pregnancy losses who were attempting to conceive. Participants were recruited from 2007 to 2011 at four United States medical centers. Participants were randomized to receive low-dose aspirin (81 mg/day) with folic acid (400 µg/day) or placebo with folic acid beginning preconception and throughout either up to six menstrual cycles or week 36 of pregnancy, if participants conceived. Both arms of the trial were included in this secondary analysis, due to the previously demonstrated null effect of low-dose aspirin on birthweight outcomes^21^.

### Ethics approval

Each site and the data coordinating center obtained approval from their Institutional Review Board (IRB), (Intermountain Healthcare IRB, Colorado Multiple IRB, University at Buffalo Health Sciences IRB, The Wright Center for Graduate Medical Education IRB, and The Emmes Corporations). All participants provided written informed consent. The trial was registered at clinicaltrials.gov (#NCT00467363). All methods were carried out in accordance with relevant guidelines and regulations.

### Serum sample assessment

Longitudinal samples of maternal serum were collected during the preconception baseline assessment, and at 4, 8, 12, 16, 20, 28, 36 and 40 weeks’ gestation (+/− one week). Samples were immediately processed and stored in −80 °C freezers until assay. Concentrations of total cholesterol, HDL-C, and TG were measured directly from serum using the Roche COBAS 6000 chemistry analyzer (Roche Diagnostics, Indianapolis, IN, USA). LDL-C was calculated using the Friedewald formula^22^, which is the standard used to assess LDL-C values in clinical settings. Fasting status was self-reported at the time of serum collection. Hemoglobin A1C (HbA1c) was measured at baseline in whole blood using a non-porous ion Exchange High Performance Liquid Chromatography (HPLC) (Tosoh BioScience, Inc., San Francisco, CA and Tokyo, Japan) on the Tosoh Automated Analyzer HLC-723G8 (CV < 1.16%).

Lipid concentration changes during pregnancy were calculated as the difference in lipid concentration from preconception to 28 weeks: [lipid concentration at week 28 – lipid concentration at preconception visit]. The 28-week timepoint was chosen to capture change in lipid concentration from preconception to ‘peak’ concentration, which generally occurs in the third trimester^23^. Furthermore, utilization of the 28 weeks’ gestation sample as our late pregnancy timepoint avoids potential bias introduced by preterm deliveries occurring prior to the 36-week measurement. A sensitivity analysis assessing change in HDL-C concentration from preconception to 20 weeks was conducted because HDL-C concentration peaks earlier in gestation than the other lipids^23^.

### Birthweight outcomes

Birthweight was obtained from neonatal delivery chart abstraction. The primary outcomes were small-for-gestational age (SGA) and large-for-gestational age (LGA) neonate at birth, defined as less than 10^th^ percentile or greater than 90^th^ percentile birthweight, respectively, for neonatal sex and gestational age at delivery using a birthweight reference^24^. Birthweight z-score was derived as a secondary outcome.

### Maternal characteristics

Maternal characteristics were assessed at baseline (preconception). Height, weight, waist circumference, and hip circumference were measured; BMI was calculated from measured height and weight at baseline. Total gestational weight gain was calculated using measured weight at the last visit before delivery (about 36 weeks gestation) and measured preconception weight at baseline. Demographic information, including maternal age, race, lifestyle habits, education level and income, was collected via questionnaire.

### Statistical analysis

Women who became pregnant with a singleton gestation and delivered between 22 and 43 weeks (within range of the reference used to derive birthweight outcomes) were included. Gestational age was determined by an ultrasonogram conducted in early pregnancy (mean 6.9 weeks of gestation; standard deviation (SD) 1.1) for 97% of clinically confirmed pregnancies among women who completed the trial; for the remaining 3% pregnancies, gestational age was determined using menstrual cycle dating from home-based fertility monitors provided by the study. Participant demographic characteristics, preconception and third trimester (28 weeks gestation) lipid concentrations, and change in lipid concentration from preconception to third trimester were compared between women with underweight or normal weight pre-pregnancy BMI (<25 kg/m^2^) and those with overweight or obese pre-pregnancy BMI (≥25 kg/m^2^). Henceforth, these will be referenced as the <25 BMI and ≥25 BMI groups. Differences between BMI groups were evaluated using chi-square or one-way analysis of variance for categorical or continuous data, respectively. Distributions of the individual lipid components were approximately normal.

Longitudinal lipid measurements were used to estimate the individual lipid component trajectories for total cholesterol, LDL-C, HDL-C and TG, stratified by BMI group. Linear mixed models with cubic splines and three knot points (25^th^, 50^th^, and 75^th^ percentiles), chosen to evenly divide the gestational age distribution, were used to estimate population average trajectories for each component. For this trajectory analysis using linear mixed models, log-transformed lipid measurements were used to improve model fit. Overall differences in lipid component trajectories between the two BMI groups were tested using a likelihood-ratio test with significance at P < 0.05. Because the global tests were significant for each lipid component, weekly pairwise differences were tested using Wald tests for each week from preconception to 40 weeks gestation. Weekly pairwise tests were conducted on the estimated trajectories. Multiple imputation (m = 10) was used to address missing covariates for the adjusted weekly models. In a secondary analysis, the trajectory model was repeated using three BMI groups: <25, 25–30, and >30 kg/m^2^, to assess how changes in maternal lipid profile vary by overweight and obese BMI group.

Logistic regression estimated the odds of LGA or SGA neonate in relation to preconception lipid concentration or lipid concentration change from preconception to 28 weeks, with individual lipids modeled independently per 10 mg/dL increase. Odds of LGA or SGA in relation to lipid concentration at 28 weeks was assessed separately to put findings in the context of the literature. As a secondary outcome, generalized linear models estimated the association between lipid concentration or lipid concentration change and birthweight z-score. Because cardiometabolic health could manifest as abnormally high or low lipid concentrations, preconception lipid concentrations were also tested for a potential non-linear association using restricted cubic splines.

Due to previously demonstrated differences in lipid profiles and birthweight outcomes by BMI group, all analyses were tested for effect modification by BMI group (<25 v. ≥25), using α = 0.20 as the threshold for significance^25^. All models were adjusted for the following characteristics, identified *a priori* based on biologic plausibility and previous studies: maternal age, race, education level, cigarette and alcohol use in the year prior to conception, pre-pregnancy BMI, fasting status at baseline serum collection, and total cholesterol (except those models with total cholesterol as the exposure). For models assessing change in lipid concentration, total cholesterol was replaced by average total cholesterol for the two visits. Because fasting status was not consistent longitudinally, comparison of lipid trajectories was not adjusted for fasting. For logistic regression and generalized linear models assessing birthweight outcomes in relation to change in lipid concentration, only fasting status at preconception serum collection was included as a covariate because 94.4% of subjects reported non-fasting at the 28-week serum collection. Race was excluded from the LGA logistic regression models stratified by BMI and limited to the <25 BMI group because there were no non-white women in this cohort with BMI <25 and LGA neonate. In a sensitivity analysis, regression models were repeated with the addition of parity and preconception HbA1c as covariates.

Multiple imputation (m = 10) was used for all logistic regression analyses and generalized linear models to address missing covariates and lipid measurements. Individual lipid concentrations were imputed in independent models to avoid potential issues with collinearity, and all covariates and outcomes were included in the imputation models. Because birthweight outcomes are conditional on achieving pregnancy through 22 weeks, inverse probability weights were used in all logistic regression models to account for potential selection bias^26^.

All analyses were conducted using SAS version 9.4 (SAS Institute Inc., Cary, North Carolina) or R (version 3.4.4).

## Results

Of 1,228 women enrolled in the study, 732 participants achieved an ultrasound confirmed pregnancy. Among these 732 gravidas, 7 were excluded on the basis or twin or other multiple gestation, 125 experienced a clinical loss, 8 withdrew from the trial, and 7 were missing data on gestational age at delivery or birthweight, for a total of 585 women who achieved a singleton pregnancy and delivered with gestational age and birthweight data available and 581 who delivered at a gestational age within range of the birthweight reference (22–43 weeks) used to derive outcomes. After excluding participants with missing data on pre-pregnancy BMI, there were a total of 575 gravidas included in the analyses. Of the 575 births, 62 (10.8%) were classified as LGA and 41 (7.1%) classified as SGA. More women in the ≥25 BMI group had LGA neonates (14.6%) than in the <25 BMI group (8.0%), whereas the incidence of SGA neonate was comparable for both groups (6.7% ≥25 BMI and 7.5% <25 BMI). Mean gestational age at delivery was 38w6d (SD, 1w4d), mean birthweight 3338 g (SD, 498 g), and mean birthweight z-score 0.14 (SD, 0.93). All individuals delivered after 28 weeks, the latest timepoint from which lipid measurements were used in analysis.

**Figure 1:**
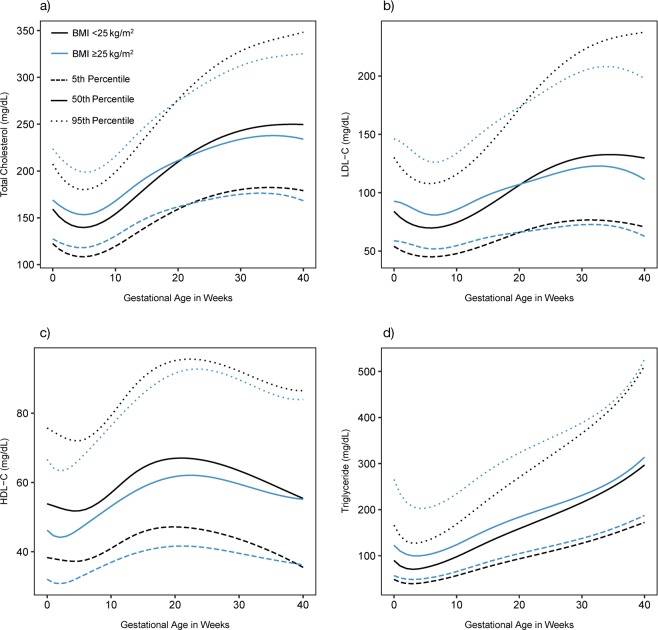
Trajectories of maternal lipid concentration (mg/dL) from preconception through 40 weeks gestation, by two preconception BMI groups in the EAGeR population. Estimated mean lipid concentration by preconception BMI group (<25 or ≥25 kg/m^2^), as estimated from linear mixed models with log-transformed outcomes and cubic splines. Limited to n = 575 women with birthweight outcomes. (**a**) Total cholesterol, (**b**) HDL-C, (**c**) LDL-C, (**d**) Triglyceride. BMI, body mass index; LDL-C, low-density lipoprotein cholesterol; HDL-C, high-density lipoprotein cholesterol.

Among women in the <25 BMI group, 17 had underweight preconception BMI (BMI <18.5), and 318 had normal weight BMI (BMI 18.5–25). Among women in the ≥25 BMI group, 136 had overweight preconception BMI (BMI 25–30), and 104 had obese BMI (BMI >30). Women in the ≥25 BMI group were more likely to have higher preconception HbA1c and lower gestational weight gain, were less likely to have education beyond high school, and were more likely to report fasting at blood draw (Table 1). Lipid trajectories varied by BMI group <25 and ≥25 (P < 0.001 for all, Fig. 1), and by BMI group <25, 25–30, and >30 (P < 0.001 for all, see Supplementary Fig. S1). Lipid trajectory analyses showed that the 25–30 and >30 groups follow a similar pattern of deviance from the <25 group, with a slightly greater magnitude of deviance for the >30 group (see Supplementary Fig. S1). Lipid concentrations and changes during pregnancy by BMI group are presented in Table 2. Women in the ≥25 BMI group had higher mean concentrations of total cholesterol, LDL-C, and TG and lower mean concentration of HDL-C at preconception compared with the <25 BMI group (pairwise p < 0.001 for all). Although the ≥25 BMI group exhibited more pronounced changes in HDL-C from preconception to 28 weeks, mean HDL-C was lower in the ≥25 BMI group compared to <25 BMI at 28 weeks (pairwise p < 0.001), consistent with preconception. The ≥25 BMI group exhibited less pronounced changes in other lipids from preconception to 28 weeks. Mean TG concentration was higher in the ≥25 BMI group at 28 weeks (pairwise p = 0.002), consistent with preconception comparison between groups. In contrast, mean LDL-C and total cholesterol concentrations in the <25 BMI group surpassed ≥25 BMI group concentrations, with statistically significantly higher concentrations in the <25 BMI group starting at 25 weeks (LDL-C, pairwise p = 0.04) or 26 weeks (total cholesterol, pairwise p = 0.04) which remained higher through the end of gestation.

**Table 1:** Maternal characteristics by BMI group.

	Overall, N = 575	Preconception BMI <25 kg/m^2^, N = 335	Preconception BMI ≥25 kg/m^2^, N = 240	*P* value
Maternal age, mean (SD)	28.5 (4.5)	28.6 (4.5)	28.5 (4.6)	0.877
Preconception BMI, mean (SD)	25.4 (5.9)	21.6 (2.0)	30.6 (5.5)	<0.0001
Preconception HbA1c, mean (SD)	5.0 (0.3)	5.0 (0.3)	5.1 (0.3)	0.003
Gestational weight gain mean (SD)	12.7 (5.5)	13.4 (4.7)	11.7 (6.4)	0.001
Race				0.652
White	558 (97.0%)	326 (97.3%)	232 (96.7%)	
Non-white	17 (3.0%)	9 (2.7%)	8 (3.3%)	
Education				0.006
Beyond high school	515 (89.6%)	310 (92.5%)	205 (85.4%)	
Up to high school	60 (10.4%)	25 (7.5%)	35 (14.6%)	
Alcohol use in the past year				0.577
Never	386 (67.1%)	228 (68.1%)	158 (65.8%)	
Sometimes	182 (31.7%)	103 (30.7%)	79 (32.9%)	
Missing	7 (1.2%)	4 (1.2%)	3 (1.3%)	
Cigarette use in the past year				0.398
Never	520 (90.4%)	306 (91.3%)	214 (89.2%)	
Sometimes	53 (9.2%)	28 (8.4%)	25 (10.4%)	
Missing	2 (0.4%)	0 (0%)	0 (0%)	
Fasting status at preconception				0.027
Fasting	70 (12.2%)	32 (9.6%)	38 (15.8%)	
Non-fasting	498 (86.6%)	297 (88.7%)	201 (83.8%)	
Missing	7 (1.2%)	6 (1.8%)	1 (0.4%)	
Parity				
Nulliparous	235 (40.9%)	140 (41.8%)	95 (39.6%)	0.595
Parous	340 (59.1%)	195 (58.2%)	145 (60.4%)	

Maternal characteristics for the overall cohort and by BMI group (<25 v. ≥25 kg/m^2^), limited to n = 575 women with singleton birth between 22–43 weeks and birthweight and maternal preconception BMI available. Values are n (%) unless otherwise specified. *P* value for difference in characteristic by BMI group (one-way ANOVA or Chi-square) is presented.

BMI, body mass index; HbA1c, hemoglobin A1C.

**Table 2:** Maternal lipid concentrations and gestational change in lipid concentrations by BMI group.

	Overall, N = 575	Preconception BMI <25 kg/m^2^, N = 335	Preconception BMI ≥25 kg/m^2^, N = 240	*P* value
Preconception lipid concentration^a^ (mg/dL)				
Total cholesterol	165.4 (28.6)	161.4 (27.3)	170.9 (29.5)	<0.0001
LDL-C	90.7 (25.2)	87.0 (22.8)	95.9 (27.5)	<0.0001
HDL-C	52.0 (12.3)	55.2 (12.3)	47.6 (10.7)	<0.0001
Triglyceride	114.1 (60.0)	97.2 (41.8)	137.8 (72.4)	<0.0001
28-week lipid concentration^b^ (mg/dL)				
Total cholesterol	239.8 (41.7)	243.3 (42.6)	234.9 (39.9)	0.020
LDL-C	130.7 (38.3)	134.0 (38.4)	126.1 (37.9)	0.020
HDL-C	64.5 (15.0)	66.3 (14.9)	62.1 (15.0)	0.002
HDL-C at 20 weeks^c^ (HDL-C peak)	66.6 (14.9)	69.0 (14.6)	63.4 (14.7)	<0.0001
Triglyceride	223.2 (72.8)	215.7 (71.2)	233.3 (74.5)	0.006
Change in lipid concentration, preconception to 28 weeks gestation (mg/dL)				
Total cholesterol	74.1 (38.4)	81.7 (36.7)	63.5 (38.2)	<0.0001
LDL-C	39.7 (34.7)	46.8 (33.8)	29.8 (33.5)	<0.0001
HDL-C	12.7 (11.4)	11.1 (11.4)	14.8 (11.1)	0.0002
HDL-C, 0 to 20 weeks (HDL-C peak)	14.7 (10.8)	13.8 (11.0)	15.9 (10.4)	0.027
Triglyceride	109.1 (67.1)	118.3 (65.7)	96.3 (67.0)	0.0002

Maternal lipid concentrations at preconception, at 28 weeks gestation, and the calculated change in lipid concentration from preconception to 28 weeks are presented for the overall cohort and by BMI group (<25 v. ≥25 kg/m^2^), limited to n = 575 women with singleton birth between 22–43 weeks gestation and birthweight and maternal preconception BMI available. Mean (SD) presented for all lipids. *P* value for significant difference in lipid concentration or lipid change by BMI group (one-way ANOVA) is presented.

BMI, body mass index; LDL-C, low-density lipoprotein cholesterol; HDL-C, high-density lipoprotein cholesterol.

^a^Missing lipid values, preconception: Total cholesterol and triglyceride, n = 9 overall, n = 4 BMI <25, n = 5 BMI ≥25; LDL-C, n = 16 overall, n = 7 BMI <25, n = 9 BMI ≥25; HDL-C, n = 13 overall, n = 7 BMI <25, n = 6 BMI ≥25;

^b^Missing lipid values, 28 weeks, for all lipids: n = 38 overall, n = 23 BMI <25, n = 15 BMI ≥25.

^c^Missing HDL-C values, 20 weeks: n = 36 overall, n = 23 BMI <25, n = 13 BMI ≥25.

### Preconception lipids in relation to birthweight outcomes

**Figure 2:**
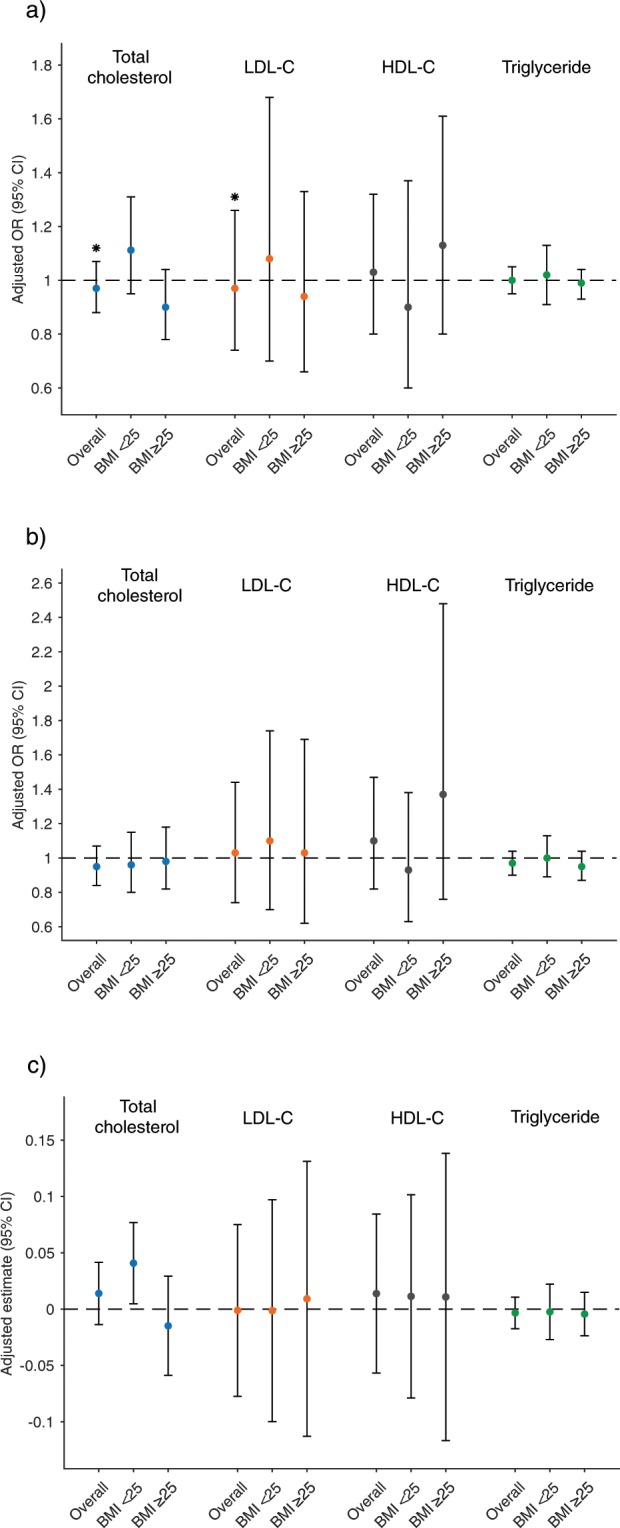
Birthweight outcomes by preconception lipid concentration and preconception BMI group. Data are interpreted as odds of large-for-gestational age (LGA) or small-for-gestational age (SGA) neonate or estimated change in birthweight z-score per 10 mg/dL increase in preconception lipid concentration (mg/dL). (**a**) Large-for-gestational age, (**b**) Small-for-gestational age, (**c**) Birthweight z-score. BMI, body mass index; LDL-C, low-density lipoprotein cholesterol; HDL-C, high-density lipoprotein cholesterol. *Indicates a significant effect modification (*P* < 0.20) by preconception BMI group (<25 kg/m^2^ v. ≥25 kg/m^2^). All models adjusted for: maternal age, race, education level, preconception smoking and alcohol habits, preconception BMI, total cholesterol (excluding total cholesterol models), and fasting status at preconception serum collection.

**Figure 3:**
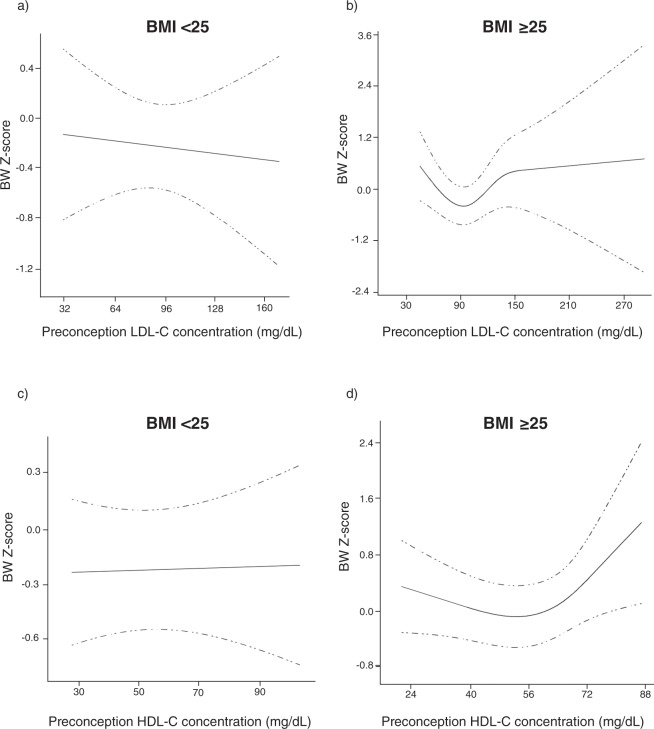
Spline regression between preconception lipid concentration (mg/dL) and birthweight z-score by preconception BMI group (<25 or ≥25 kg/m^2^). (**a**) LDL-C, BMI <25; (**b**) LDL-C, BMI ≥25; (**c**) HDL-C, BMI <25; (**d**) HDL-C, BMI ≥25. BMI, body mass index; LDL-C, low-density lipoprotein cholesterol; HDL-C, high-density lipoprotein cholesterol.

**Figure 4:**
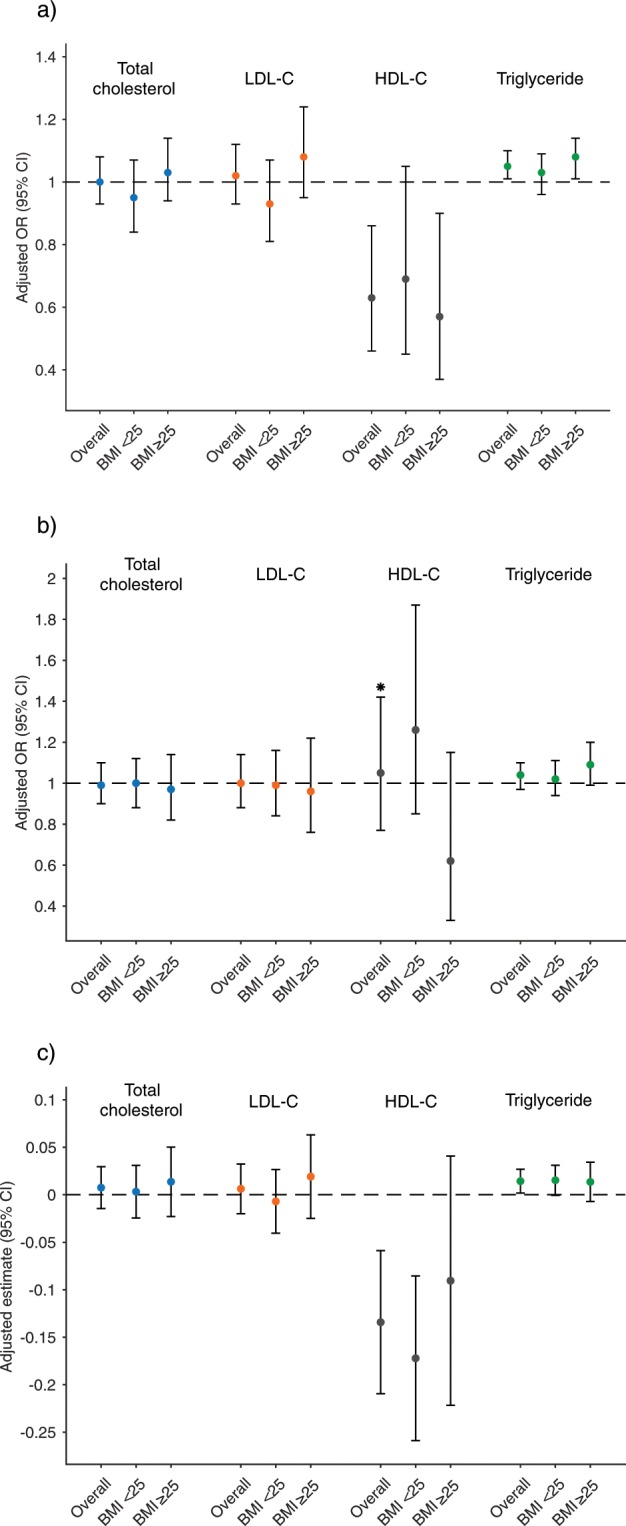
Birthweight outcomes by change in lipid concentration from preconception to 28 weeks gestation and preconception BMI group. Data are interpreted as odds of large-for-gestational age (LGA) or small-for-gestational age (SGA) neonate or estimated change in birthweight z-score per 10 mg/dL increase in lipid concentration (mg/dL) change. (**a**) Large-for-gestational age, (**b**) Small-for-gestational age, (**c**) Birthweight z-score. BMI, body mass index; LDL-C, low-density lipoprotein cholesterol; HDL-C, high-density lipoprotein cholesterol. *Indicates a significant effect modification (*P* < 0.20) by preconception BMI group (<25 kg/m^2^ v. ≥25 kg/m^2^). All models adjusted for: maternal age, race, education level, preconception smoking and alcohol habits, preconception BMI, average total cholesterol of preconception and 28-week visit (excluding total cholesterol models), and fasting status at preconception serum collection.

There were no associations between preconception lipid concentrations and odds of LGA or SGA neonate in the fully adjusted models (Fig. 2). There was a potential U-shaped relationship between preconception LDL-C and birthweight z-score (test for curvature [significance indicates evidence of non-linearity], p < 0.001; overall significance of the curve [significance indicates an overall association between lipids and birthweight outcomes], p = 0.003) and HDL-C (test for curvature, p = 0.021; overall significance of the curve, p = 0.098) in the ≥25 BMI group only, with both high and low preconception concentrations of HDL-C and LDL-C associated with increased birthweight z-score although the estimates were imprecise at the extremes (Fig. 3). Effect modification by BMI group was observed in at least one birthweight outcome regression model for all lipids except TGs, so results are presented both overall and stratified by BMI group for all lipids, with significant interactions (P < 0.20) denoted (Figs. 2–4).

### Lipids changes during pregnancy in relation to birthweight outcomes

Changes in lipid concentration during pregnancy in relation to birthweight outcomes are presented in Fig. 4. A 10 mg/dL increase in HDL-C concentration change from preconception to 28 weeks was associated with 37% decreased odds of LGA neonate overall (odds ratio (OR) = 0.63, 95% confidence interval (CI): 0.46, 0.86). A 10 mg/dL increase in TG concentration change from preconception to 28 weeks was associated with 5% increased odds of LGA overall (OR = 1.05, 95% CI: 1.01, 1.1). Effect modification by BMI group was not observed for lipid concentration changes from preconception to 28 weeks, except for HDL-C change from preconception to 28 weeks and odds of SGA: a 10 mg/dL increase in change in HDL-C concentration from preconception to 28 weeks was associated with 38% decreased odds of SGA in the ≥25 BMI group (OR = 0.62, 95% CI: 0.33, 1.15) but not in the <25 BMI group (OR = 1.26, 95% CI: 0.85, 1.87). Cross-sectional HDL-C and TG concentration at 28 weeks followed the same association with LGA and SGA as change in lipid concentration, with increased odds of LGA associated with lower HDL-C or higher TG concentration at 28 weeks, and decreased odds of SGA associated with higher HDL-C concentration at 28 weeks in the ≥25 BMI group only (data not shown). The associations between change in lipid concentration from preconception to 28 weeks and deviation from mean birthweight z-score concurred with associations with LGA, with greater HDL-C change associated with lower birthweight z-score and greater TG change with higher birthweight z-score in the overall cohort (Fig. 4).

In a sensitivity analysis repeating the main analysis with change in HDL-C from preconception to 20 weeks (around the time during gestation when HDL-C concentrations peak), effect modification by BMI group was again observed. Associations between HDL-C change to 20 weeks and birthweight outcomes were similar to associations with HDL-C change to 28 weeks, with stronger associations between HDL-C and risk of SGA in the ≥25 BMI group at 20 weeks compared to 28 weeks. A 10 mg/dL increase in HDL-C concentration change from preconception to 20 weeks was associated with 65% decreased odds of SGA neonate at delivery in the ≥25 BMI group (OR = 0.35, 95% CI: 0.19, 0.64) but not in the <25 BMI group (OR = 1.14, 95% CI: 0.75, 1.75). The association between HDL-C and decreased odds of SGA in the ≥25 BMI group was also observed for cross-sectional HDL-C concentration at 20 weeks (OR = 0.63, 95% CI: 0.40, 0.98). Results were similar with the addition of parity and preconception HbA1c to the regression models.

## Discussion

Maternal preconception lipid profile and lipid changes from preconception throughout pregnancy differed by maternal BMI. Preconception lipid concentrations were not associated with odds of LGA or SGA, although we observed a potential U-shaped relationship between preconception HDL-C and LDL-C concentrations and birthweight z-score in women with overweight or obese BMI. Change in lipid concentration during pregnancy was associated with birthweight outcomes, particularly a more pronounced increase in HDL-C, which was associated with decreased odds of LGA overall and decreased odds of SGA for women with overweight or obese BMI. Our findings indicate that lipid changes from preconception to late pregnancy are associated with birthweight outcomes, with more pronounced change in HDL-C associated with more favorable outcomes. The novel finding that changes in lipid concentration from preconception to second and third trimester differed by BMI group and were differentially associated with birthweight outcomes adds to the understanding of how lipid metabolism during pregnancy differs by maternal BMI and contributes to fetal growth.

The observed difference in lipid trajectories by BMI group are generally consistent with previous studies for total cholesterol, LDL-C, and TG changes, although others did not find differences in HDL-C change by BMI group whereas we observed more pronounced changes in HDL-C for women with pre-pregnancy BMI ≥25^16,17^. Differences could be due to the larger sample size in the present study, or because our analysis captured previously unexplored early HDL-C changes by including preconception samples. Lipid trajectory analyses with further stratifications of the ≥25 BMI group into 25–30 and >30 support a dose-response relationship of increasing gestational dyslipidemia with increasing preconception BMI.

Preconception lipid concentrations were not associated with risk of SGA or LGA, consistent with the few previous studies on preconception lipids, which found no significant association with birthweight outcomes^18,19^. However, we observed a potential U-shaped relationship between birthweight z-score and LDL-C and HDL-C concentrations in women with overweight or obese pre-pregnancy BMI, which may indicate that preconception lipids at either extreme predict altered fetal growth and birthweight. One of the prior studies tested for a non-linear relationship and observed no association; this discrepancy may be due to differences in study populations, since the other was a non-United States cohort of subjects with only 14% of participants having BMI ≥25, limiting generalizability to the more diverse range of pre-pregnancy BMI in the United States population. The observed U-shaped relationships are biologically plausible, as similar U-shaped relationships between lipid concentrations and morbidity; including risk of dementia, immune function, and cardiovascular outcomes; have been described in epidemiologic studies of non-pregnant populations^27–29^.

Lipid changes from preconception to mid- to late- pregnancy were also associated with birthweight outcomes, and these findings are somewhat difficult to put into context with prior data since most studies only evaluated lipid concentrations at a cross-sectional time points during pregnancy. In our study, associations between cross-sectional lipid concentrations at 28 weeks and birthweight outcomes followed the same pattern as our examination of the change in lipids from preconception to 28 weeks’ gestation, suggesting that cross-sectional lipid concentrations evaluated in prior studies are likely more reflective of changes during pregnancy rather than preconception lipid profile. In our analysis, high HDL-C concentration in late pregnancy was strongly associated with decreased risk of LGA birth and decreased birthweight for gestational age, consistent with previous findings^9–12,14^. When stratified by BMI group, some prior studies found a significant association only in gravidas with a pre-pregnancy BMI ≥25^9,11^, while others found an association in both BMI groups but with attenuated risk in women with BMI <25. High TG concentration in late pregnancy was associated with increased risk of LGA birth in the ≥25 BMI group, although the confidence interval was close to unity and should be interpreted with caution. Our findings indicate that low HDL-C and high TG concentrations are associated with increased risk of LGA regardless of pre-pregnancy BMI, and add the novel observation that it is the change in lipid concentration from preconception to 28 weeks that was strongly associated with birthweight outcomes, which had not been previously explored.

Interestingly, we found decreased odds of SGA neonate with a greater change in HDL-C from preconception to 20 weeks and preconception to 28 weeks in the ≥25 BMI group only. The association was strongest with change from preconception to 20 weeks, which is the time of HDL-C concentration peak during gestation. In contrast to our results, four prospective cohort studies found no association between maternal lipid profile in the second and third trimesters and risk of SGA neonate^14,30–32^, though two retrospective studies observed higher second or third trimester HDL-C concentrations in pregnancies complicated by SGA^33,34^. None of these prior studies presented results by BMI group or evaluation of lipid changes from preconception to second trimester, however, which could explain why ours is the first study to report an association between more pronounced HDL-C change over this time period and decreased risk of SGA in women with BMI ≥25. It is plausible that the change in HDL-C from preconception to mid- to late-pregnancy represents a metabolic adaptation that is supportive of optimal fetal growth that cannot be captured by cross-sectional measures in pregnancy.

The association between greater HDL-C change during gestation and decreased risk of both LGA and SGA could be explained by HDL-C’s dual functionality in regulating inflammation and maintaining cholesterol homeostasis. For example, higher HDL-C concentration during gestation is associated with decreased risk of preeclampsia and preterm birth, most likely due to the association between HDL-C and decreased inflammation, which would improve fetal-placental circulation^35,36^. Obesity is characterized by increased inflammation and poor vascular adaptation to pregnancy^37,38^, which could explain why HDL’s association with decreased risk of SGA in the present study was in the ≥25 BMI group only. Regarding cholesterol homeostasis, HDL-C is involved in the removal and transport of cholesterol from cells to the liver in non-pregnant individuals^39^, and through this mechanism could help prevent fetal overgrowth under conditions of excessive cholesterol during pregnancy.

Although the EAGeR cohort provided a relatively large sample with diverse pre-pregnancy BMI, the study was limited in terms of diversity in maternal race and ethnicity, cigarette use, education level, and age. The cohort size limited our ability to further stratify the ≥25 BMI group for regression models. In addition, all women included in the trial had experienced a prior pregnancy loss. Thus, generalizability may be limited. Furthermore, women in the cohort had relatively healthy mean preconception lipid concentrations, potentially limiting the ability to detect birthweight outcomes in association with more extreme preconception dyslipidemia. Our analysis of maternal lipids was limited to current technology widely available in clinical practice, which only captures major lipoprotein and lipid concentrations, while emerging evidence has demonstrated that the sub-fractions and density, particularly of HDL-C and LDL-C, are key factors in lipid functionality and physiologic effects.

The major strength of our study is the novel addition of changes in lipid concentration from preconception to late pregnancy in association with birthweight outcomes. The availability of longitudinal measures of lipid concentration during pregnancy allowed us to target changes from preconception to lipid concentration peak during gestation. The diversity of pre-pregnancy BMI and relatively large sample size allowed for the investigation of effect modification by BMI, which revealed that HDL-C may have a stronger effect on risk of SGA in women with BMI ≥25, possibly due to the inflammatory state associated with increased adiposity during gestation.

## Conclusion

Changes in lipid concentration from preconception to third trimester were associated with birthweight outcomes, primarily HDL-C, for which a greater rise during pregnancy was associated with decreased odds of LGA overall and decreased odds of SGA in gravidas with overweight or obese pre-pregnancy BMI. Birthweight outcomes were not strongly associated with preconception lipid profile, suggesting that the association between abnormal lipid concentrations during pregnancy and unhealthy birthweight outcomes is reflective of poor maternal adaptation to pregnancy. This study was limited to women with 1–2 prior pregnancy losses who were trying to achieve pregnancy; therefore generalizability to a broader population may be limited. Additional studies should help elucidate the mechanisms linking overweight and obese pre-pregnancy BMI with lipid changes during gestation and fetal growth and may offer insight for intervention to prevent low and high birthweight and associated adverse outcomes.

## Supplementary information

Supplementary Information

## Data Availability

The analytic file prepared for this work along with its supporting documentation is available upon request from the corresponding author consistent with the NIH Intramural Research Program’s data sharing policy.
