# Supplementary material for: Maternal preconception lipid profile and gestational lipid changes in relation to birthweight outcomes
Source: Sci Rep. 2020 Jan 28;10:1374. doi: 10.1038/s41598-019-57373-z (PMC6987205; doi:10.1038/s41598-019-57373-z)

Title: Maternal preconception lipid profile and gestational lipid changes in relation to birthweight outcomes

Running title: Maternal lipid profile and birthweight outcomes

Authors: Alaina M. Bever, B.S.[1]; Sunni L. Mumford, Ph.D.[1]; Enrique F. Schisterman, Ph.D.[1]; Lindsey Sjaarda, Ph.D.[1]; Neil J. Perkins, Ph.D.[1]; Nicole Gerlanc, Ph.D.[2]; Elizabeth A. DeVilbiss, Ph.D.[1]; Robert M. Silver, M.D.[3]; Keewan Kim, Ph.D.[1]; Carrie J. Nobles, Ph.D.[1]; Melissa M. Amyx, Ph.D.[1], M.P.H.; Lindsay D. Levine, B.A.[1]; Katherine L. Grantz*, M.D., M.S.[1]

Affiliations:

[1] Epidemiology Branch, Division of Intramural Population Health Research, *Eunice Kennedy Shriver* National Institute of Child Health and Human Development, 6710B Rockledge Drive, MSC 7004, Bethesda, MD 20892, United States.

[2] The Prospective Group, 1655 Fort Myer Dr #700, Arlington, VA 22209, United States.

[3] Department of Obstetrics and Gynecology, University of Utah, 30 North 1900 East, Salt Lake City, UT 84132, United States.

Contact information for corresponding author:

Dr. Katherine L. Grantz, M.D., M.S., Investigator, Epidemiology Branch, DIPHR, NICHD, NIH, 6710B Rockledge Drive, MSC 7004, Bethesda, MD 20892, Tel: 1-301-435-6935, Fax: 301-402-2084

Email: katherine.grantz@nih.gov

Figure Legends

Supplementary Figure 1. Trajectories of maternal lipid concentration (mg/dL) from preconception through 40 weeks gestation, by three preconception BMI groups in the EAGeR population. Estimated mean lipid concentration by preconception BMI group (<25, 25-30, or >30 kg/m^2), as estimated from linear mixed models with log-transformed outcomes and cubic splines. Limited to n=575 women with birthweight outcomes. A) Total cholesterol, B) HDL-C, C) LDL-C, D) Triglyceride. BMI, body mass index; LDL-C, low-density lipoprotein cholesterol; HDL-C, high-density lipoprotein cholesterol.

Supplementary Figure 1

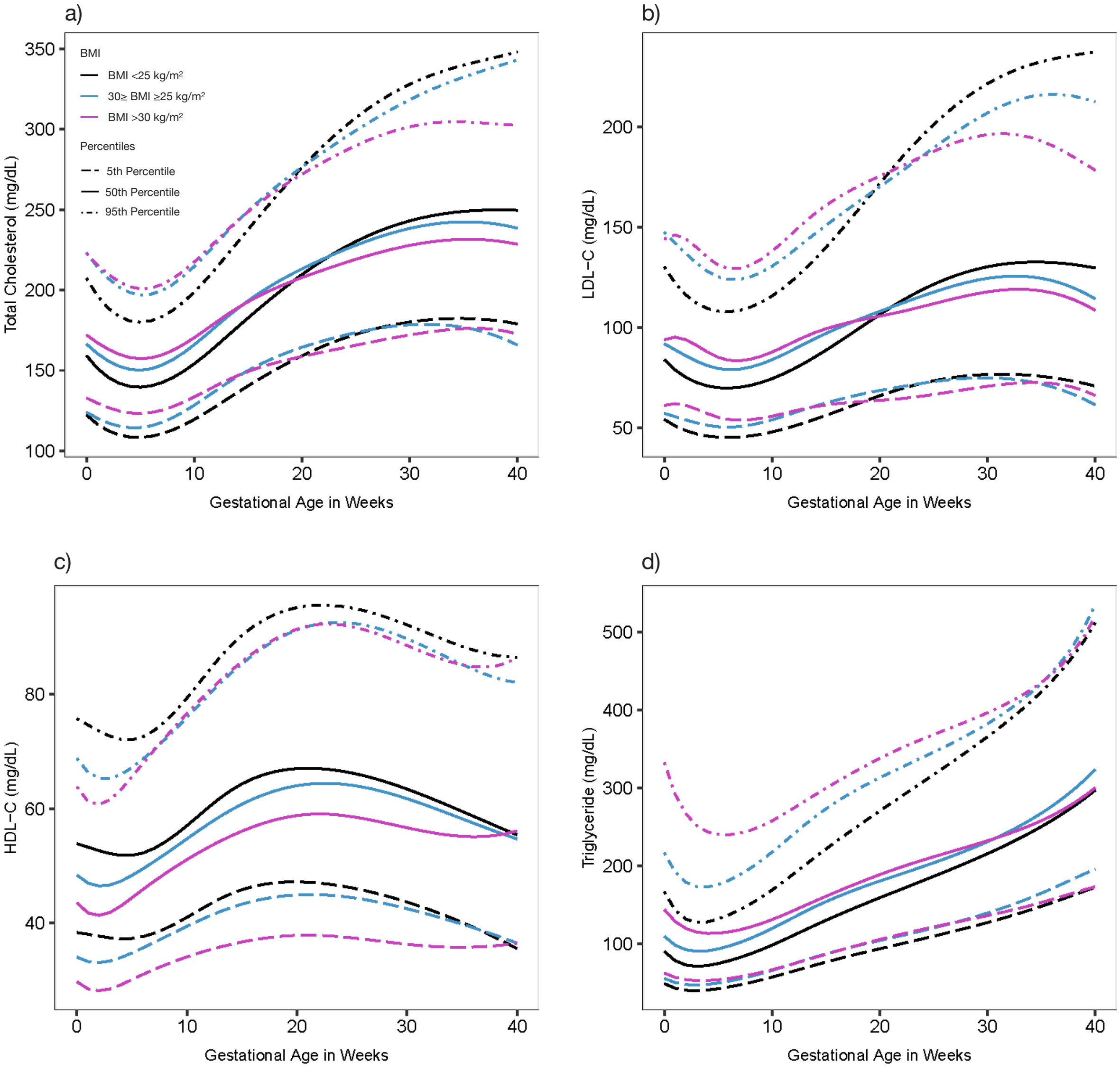

Supplement: Supplementary file 1 — Supplementary Information [file 41598_2019_57373_MOESM1_ESM.pdf]
